# Deep learning for cephalometric landmark detection: systematic review and meta-analysis

**DOI:** 10.1007/s00784-021-03990-w

**Published:** 2021-05-27

**Authors:** Falk Schwendicke, Akhilanand Chaurasia, Lubaina Arsiwala, Jae-Hong Lee, Karim Elhennawy, Paul-Georg Jost-Brinkmann, Flavio Demarco, Joachim Krois

**Affiliations:** 1 Department of Oral Diagnostics, Digital Health and Health Services Research, Charité – Universitätsmedizin Berlin, Berlin, Germany; 2 Topic Group Dental Diagnostics and Digital Dentistry, ITU/WHO Focus Group AI on Health, Berlin, Germany; 3 Department of Oral Medicine and Radiology, King George’s Medical University, Lucknow, India; 4 Department of Periodontology, Daejeon Dental Hospital, Institute of Wonkwang Dental Research, Wonkwang University College of Dentistry, Daejeon, Korea; 5 Department of Orthodontics, Dentofacial Orthopedics and Pedodontics, Charité – Universitätsmedizin Berlin, Berlin, Germany; 6 Post-Graduate Program in Epidemiology, Federal University of Pelotas, Pelotas, Brazil

**Keywords:** Artificial intelligence, Convolutional neural networks, Evidence-based medicine, Meta-analysis, Orthodontics, Systematic review

## Abstract

**Objectives:**

Deep learning (DL) has been increasingly employed for automated landmark detection, e.g., for cephalometric purposes. We performed a systematic review and meta-analysis to assess the accuracy and underlying evidence for DL for cephalometric landmark detection on 2-D and 3-D radiographs.

**Methods:**

Diagnostic accuracy studies published in 2015-2020 in Medline/Embase/IEEE/arXiv and employing DL for cephalometric landmark detection were identified and extracted by two independent reviewers. Random-effects meta-analysis, subgroup, and meta-regression were performed, and study quality was assessed using QUADAS-2. The review was registered (PROSPERO no. 227498).

**Data:**

From 321 identified records, 19 studies (published 2017–2020), all employing convolutional neural networks, mainly on 2-D lateral radiographs (n=15), using data from publicly available datasets (n=12) and testing the detection of a mean of 30 (SD: 25; range.: 7–93) landmarks, were included. The reference test was established by two experts (n=11), 1 expert (n=4), 3 experts (n=3), and a set of annotators (n=1). Risk of bias was high, and applicability concerns were detected for most studies, mainly regarding the data selection and reference test conduct. Landmark prediction error centered around a 2-mm error threshold (mean; 95% confidence interval: (–0.581; 95 CI: –1.264 to 0.102 mm)). The proportion of landmarks detected within this 2-mm threshold was 0.799 (0.770 to 0.824).

**Conclusions:**

DL shows relatively high accuracy for detecting landmarks on cephalometric imagery. The overall body of evidence is consistent but suffers from high risk of bias. Demonstrating robustness and generalizability of DL for landmark detection is needed.

**Clinical significance:**

Existing DL models show consistent and largely high accuracy for automated detection of cephalometric landmarks. The majority of studies so far focused on 2-D imagery; data on 3-D imagery are sparse, but promising. Future studies should focus on demonstrating generalizability, robustness, and clinical usefulness of DL for this objective.

**Supplementary Information:**

The online version contains supplementary material available at 10.1007/s00784-021-03990-w.

## Introduction

Medical applications using artificial intelligence (AI) are increasingly common; one of the most prolific fields in this regard is computer vision, i.e., AI-based image analysis. Deep learning (DL), a subfield of machine learning, especially DL using convolutional neural networks (CNNs) has been demonstrated to be highly suitable for computer vision. One of the most common strategies in machine learning is supervised learning, where an algorithm is exposed to pairs of data and data labels (e.g., for computer vision, an image, and the corresponding image label). During the model “training” phase, these data pairs are repeatedly shown to the algorithm by which the DL model (specifically the model weights) is iteratively optimized to minimize the error in the model predictions. A well-trained DL model learnt to represent the (nonlinear) statistical structure of the input data and its relation to the given label [1] and is eventually capable to predict a label on new, i.e., unseen data (images).

A range of relevant aspects when training and testing DL models (e.g., CNNs) for medical applications have been identified [2–4]: (1) The representativeness of the training and test datasets needs to be ensured if generalizability of the model is expected. (2) Labelling of images is complex, as there is seldom one hard “gold standard” (e.g., histological assessment) available; more often, multiple human experts label the same image, and a range of options to unify these “fuzzy” labels have been used [5]. Different strategies to establish a reliable gold standard are available, e.g., majority voting schemes. (3) The value of any model should be demonstrated, for example, by presenting its performance against that of the current standard of care (e.g., individual healthcare providers) on a separate test dataset. Presenting the performance of a model on the same dataset it learnt from will yield highly inflated performance metrics [6].

Cephalometric radiographs are taken by orthodontists to quantitatively evaluate the skeletal relationship between the cranial base and the maxilla or mandible, the relationship between maxilla and mandible, and the dentoalveolar relationship. They also serve for determining the growth pattern through quantitative and qualitative evaluations and superimposition of serial radiographs [7]. Moreover, cephalograms are required for planning orthognathic surgery [8, 9]. A key task on such cephalometric 2-D radiographs or 3-D CT or cone beam CT (CBCT) images is landmark detection. While the value of cephalometric analysis and the definition of landmarks remains an issue of debate [10], automating this task has been identified as useful, particularly as landmarking is laborsome, requiring the time of experienced (and expensive) experts [11, 12]. Automated landmark detection for cephalometric analysis has been in the focus for decades, while DL has been demonstrated to possibly exceed less advanced (e.g., knowledge-based or atlas-based) systems [13]. Moreover, DL-based cephalometric software applications from different companies (e.g., CellmatIQ, Hamburg, Germany; ORCA AI, Herzliya, Israel; WebCeph, Gyeonggi-do, Korea) are by now available to orthodontists worldwide.

The number of studies involving DL for landmark detection on 2-D and 3-D cephalometric imagery is increasing rapidly, while it remains uncertain how robust and consistent the emerging body of evidence is. Moreover, it is unclear if the accuracy of DL or the quality of the studies is improving over time, or if there are differences in accuracy on 2-D versus 3-D imagery. The present systematic review and meta-analysis evaluated studies employing DL for landmark detection on cephalometric 2-D or 3-D radiographs. Our research question was as follows: What is the accuracy of DL for detecting landmarks on cephalometric radiographs?

## Materials and methods

Reporting of this review and meta-analysis followed the PRISMA checklist [14]. The study protocol was registered after the initial screening stage (PROSPERO registry no. 227498). Our PICO question was as follows: In 2- or 3-D radiographic imagery suitable for cephalometric landmark detection (participants), comparing DL (intervention) versus conventional landmarking of individual experts or against a gold standard (e.g., of multiple experts) (control), what is the accuracy (outcome)?

### Eligibility criteria

The following selection criteria were applied: (1) diagnostic accuracy studies employing DL, e.g., CNNs; (2) trained and tested on 2-D- or 3-D cephalometric imagery like 2-D lateral or frontal radiographs or 3-D CT or CBCT, with minimum 5 relevant landmarks to be detected and sufficient detail to extract information on the train and test dataset sizes; (3) reporting their outcome as the mean deviation from a 2-mm prediction error threshold (e.g., studies reporting their accuracy to be below or above this threshold) (including mean and variance) or the proportion of landmarks correctly predicted within this 2-mm prediction error threshold; and (4) published 2015–2020, as we did not expect DL studies in this field to be published before that (mainly as DL was not available much earlier and the first applications in medicine evolved since 2015), in English. Only studies fulfilling all of the above-described criteria were included. Studies on non-radiographic data, cephalometrically irrelevant landmarks (e.g., those of the brain), or using non–deep learning methods (e.g., knowledge- or atlas-based or involving shallow machine learning) were excluded.

### Information sources and search

We systematically screened four electronic databases (Medline via PubMed, Embase via Ovid, IEEE Xplore, arXiv) for studies published up January 2015 to December 2020. Medline and Embase are widely used and partially complementary medical databases. IEEE (Institute of Electrical and Electronics Engineers) Xplore is a library for articles, proceedings, and standards in physics, computer science, engineering, and related fields, indexing 200+ journals and 3+ million conference papers. arXiv is an archive of electronic preprints for research articles of scientific topics such as physics, mathematics, computer science, and statistics. Archived articles may be published later in more traditional journals, and while arXiv is not peer reviewed, there are moderators who review the submissions [15]. The search was overall designed to account for different publication cultures across disciplines.

**Fig. 1:**
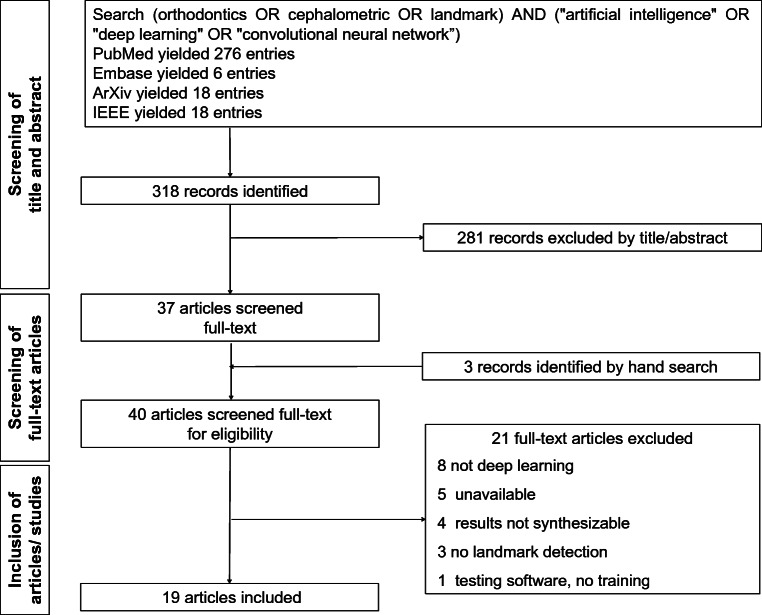
Flowchart of the search

A two-pronged search strategy, combining the technique of interest (AI, CNN, DL, etc.) and the diagnostic target (landmark detection, cephalometry, orthodontics), was applied. The search sequence was adapted for each database/repository, an example for Medline can be found in Fig. 1. Reviews or editorials were excluded, and cross-referencing from bibliographies was performed.

### Screening

Screening of titles or abstracts was independently performed by two reviewers (FS, AC). Any disagreement was resolved by discussion. All papers which were found to be potentially eligible were assessed in full text against the inclusion criteria. Inclusion and exclusion were decided by two reviewers in consensus (FS, AC).

### Data collection, items, and preprocessing

A pretested spreadsheet was used to collect data. Study characteristics, including country, year of publication, imagery (2-D lateral or frontal radiographs or 3-D imagery), dataset source, size and partitions (training, test), characteristics/architecture of the DL strategy used, number of landmarks detected, reference test and its unification in case of multiple annotators, comparators (current standard of care, e.g., clinicians), accuracy metrics, and findings, were extracted by two reviewers (FS, AC). If one study reported on several models or test datasets, these were extracted.

### Quality assessment

Risk of bias was assessed using the QUADAS-2 tool [16], accounting for bias in the data selection (no inappropriate exclusions, no case-control design, random or consecutive inclusion), index test (assessment blinded for and independent of reference test), reference test (valid reference test, assessment independent from index test), flow and timing (sufficient time between index and reference, all datapoints included in analysis), as well as applicability concerns for the data (data match review question), index test (test, conduct, and interpretation match review question), and reference test (the way landmarks were established matches review question). Risk of bias was assessed independently by two reviewers, who discussed their findings in case of disagreement to come to a consensus. We do not provide further guidance as to the certainty of the evidence (e.g., using any kind of grading), but provide descriptive statistics of the individual and overall risk of bias together with meta-analytic estimates.

### Summary measures and data synthesis

The criteria for a study to be included into meta-analysis was that it reported one of our two accuracy outcomes, the deviation from a 2-mm prediction error threshold (in mm) or the proportion of landmarks correctly predicted within this 2-mm prediction error threshold (reported as mean and measures of variance and/or sample size, allowing weighing of the study estimates). Our summary measures were the mean deviation from the 2-mm threshold (in mm; only one study reported this in another metric, namely pixels, and was therefore excluded) or the proportion of landmarks predicted within this 2-mm threshold, both with their 95% confidence intervals (CI). Heterogeneity was assessed using Cochrane’s Q and I^2^ statistics [17]. Random-effects models were used for meta-analysis; the statistical package metaphor [18], implemented in OpenMetaAnalyst [19], was employed. To allow weighting of studies for the synthesis of proportions, we recalculated the number of true predictions (within the 2-mm threshold) in the overall test dataset, accounting for its size and the number of landmarks predicted in each study. If studies reported on multiple test datasets, we handled them as independent units, accounting for this multiplicity of accuracy data. To explore reasons for heterogeneity, we performed subgroup analyses and mixed-effect meta-regression. For the latter, the unrestricted maximum likelihood method was used.

## Results

### Study selection and characteristics

From 321 identified studies, 40 were screened in full texts, and 19 studies were eventually included in our review and meta-analysis (Fig. 1). The 21 excluded studies, with reasons for exclusion, can be found in Table S1. The included studies (Table 1) were published between 2017 and 2020 (median: 2020) and stemmed from nine countries (Korea, 7 studies; China, 4 studies; Japan, 2 studies; all six remaining countries, 1 study). Fifteen studies focused on the analysis of 2-D radiographs, four on 3-D radiographs. Eleven studies used publicly available data from the IEEE 2015 grand challenge [39]; one of these also used own data. A second publicly available dataset (CQ 500; http://headctstudy.qure.ai/dataset) was used, together with own data, by one study. Seven studies used own data; one study did not report on the dataset in detail.

**Table 1:** Included studies

1st author	Year	Country	Imagery	Data source	Architecture/modelling framework	N landmarks	Total sample	Train/validate sample	Reference test on training/validation data	Unification of labels	Test sample	Reference test on test data	Unification of labels
Arik 2017 [20]	2017	USA	Lateral 2D	IEEE Grand Challenge 2015	Custom CNN combined with a shape model for refinement	19	400	150	2 experts	Average	150+100	2 experts	Average
Chen 2019 [21]	2019	China	Lateral 2D	IEEE Grand Challenge 2015	VGG-19, ResNet20, and Inception; custom attentive feature pyramid fusion module	19	400	150	2 experts	Average	150+100	2 experts	Average
Gilmour 2020 [22]	2020	Canada	Lateral 2D	IEEE Grand Challenge 2015	Modified ResNet34 combined with a custom image pyramids approach (spatialized features)	19	400	150	2 experts	Average	150+100	2 experts	Average
Huang 2020 [23]	2020	Germany	Lateral 2D	CQ500 CTs (train) and IEEE Grand Challenge 2015 (test)	LeNet-5 for ROI patches and ResNet50 for landmark location	19	na	491	3 radiologists	Majority	150	2 experts	Average
Hwang 2020 [24]	2020	Korea	Lateral 2D	Own dataset	Customized YOLO V3	80	1311	1028	1 expert	NA	283	1 expert	NA
Kim 2020 [25]	2020	Korea	Lateral 2D	Own dataset+IEEE Grand Challenge 2015	Stacked hourglass-shaped networks	23	2475	1875	2 experts	Unclear	200+225+400	2 experts	Unclear or average (IEEE)
Lee 2020 [26]	2020	Korea	Lateral 2D	IEEE Grand Challenge 2015	Custom CNN for ROI and custom Bayesian CNN for landmark detection	19	400	250	2 experts	Unclear	150	2 experts	Average
Lee 2019 [27]	2019	Japan	Lateral 2D	Own dataset	Combined custom CNNs for ROI classification and point estimation	22	936	835	3 experts	Unclear	100	3 experts	Unclear
Lee 2019 [28]	2019	Korea	3D	Own dataset	VGG-19	7	27	20	2 experts	Average	7	2 experts	average
Ma [29]	2020	Japan	3D	Own dataset	Custom CNNs for classification and regression	13	66	58	1 expert	NA	8	1 expert	NA
Muraev 2020 [30]	2020	Russia	Frontal 2D	Unclear	Multiclass FPN and ResNeXt-50 with Squeeze-and-Excitation blocks	45	330	300	Students, corrected by experts	Consensus	30	students, corrected by experts	Consensus
Noothout 2020 [31]	2019	Netherlands	Lateral 2D	IEEE Grand Challenge 2015	Custom FCNs based on ResNet34	19	400	150	2 experts	Unclear	150+100	2 experts	Average
O'Neil 2018 [32]	2018	UK	Lateral 3D	Own dataset	Custom FCN and Atlas Correction	22	22	201	3 experts	Unclear	20	2 experts	Unclear
Oh 2020 [33]	2020	Korea	Lateral 2D	IEEE Grand Challenge 2015	DACFL, custom FCN combined with a local feature perturbator and the anatomical context loss	19	400	150	2 dental experts	Average	150+100	2 experts	Average
Park 2020 [34]	2019	Korea	Lateral 2D	Own dataset	YOLO V3 and SSD	80	1311	1028	1 expert	NA	283	1 expert	NA
Qian 2020 [35]	2020	China	Lateral 2D	IEEE Grand Challenge 2015	Cepha-NN, combining U-Net-shaped networks, attention mechanism, and region enhancing loss	19	400	150	2 experts	Average	150+100	2 experts	Average
Song 2020[36]	2020	China	Lateral 2D	IEEE Grand Challenge 2015	ROI extraction and ResNet50	19	400	150	2 experts	Average	150+100	2 experts	Average
Yun 2020 [37]	2020	Korea	3D	Own dataset	Custom CNNs, combined skull normalization, and VAE for coarse to fine detection tasks	93	26	22	1 expert	NA	4	1 expert	NA
Zhong 2020 [38]	2019	China	Lateral 2D	IEEE Grand Challenge 2015	2-stage (global and local) U-Net models	19	400	150	2 experts	Average	150+100	2 experts	Average

Abbreviations: FCN, fully convolutional neural network; ROI, region of interest. Single Shot Detector.

All studies employed CNNs, with VGG-19 (n=2) and YOLO V3 (n=2), Resnet50 (n=2) and ResNet34 (n=2) being the most frequent architectures. The studies tested the detection of a mean of 30 (SD: 25; range: 7–93) landmarks. The size of the training dataset was 479 in mean (150; 20–1875); the size of the test dataset was 128 (83; 4-283).

The reference test in the training dataset was established by two experts in 11 studies, 1 expert in four studies, and 3 experts in three studies; one study used students to label the landmarks and had these corrected by experts. Eight studies used the average to come to a unified label when having more than one annotator, one used a consensus process, one used a majority voting scheme, and five studies did not report on that; for the studies with only 1 annotator, this was not relevant. The reference test in the test dataset was by large established similarly (2 experts: n=13; 1 expert: n=4; 3 experts: n=1; corrected students: n=1). Notably, many studies employed multiple test datasets, mainly as the IEEE 2015 Grand Challenge included two test datasets, one with 150 and one with 100 cephalometric images.

### Risk of bias and applicability concerns

Risk of bias was assessed in four domains and found high for most studies regarding the data selection (n=16), reference test (n=18), but not index test (n=7) or flow and timing (n=1). Applicability concerns were present for most studies toward the data selection (n=16), reference test (n=18), but not index test (n=8). A detailed assessment of risk of bias and applicability concerns can be found in Table 2.

**Table 2:**
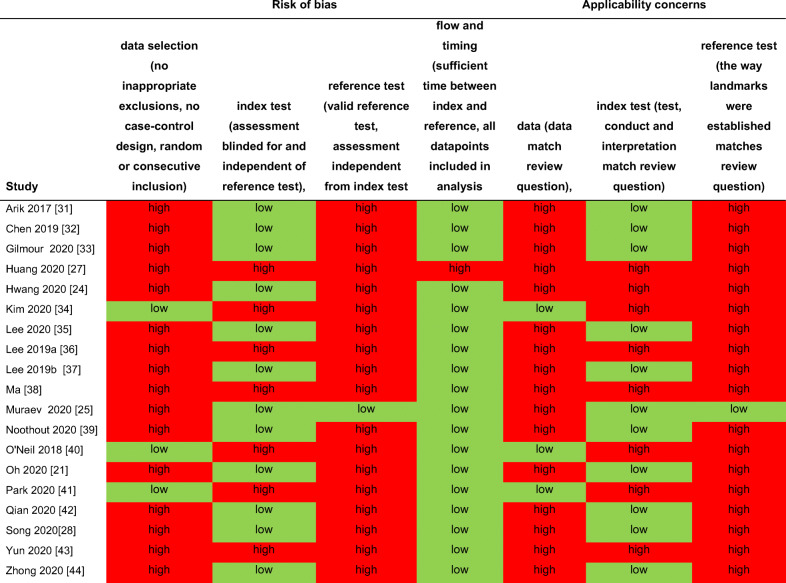
Risk of bias and applicability concerns

### Meta-analysis

**Fig. 2:**
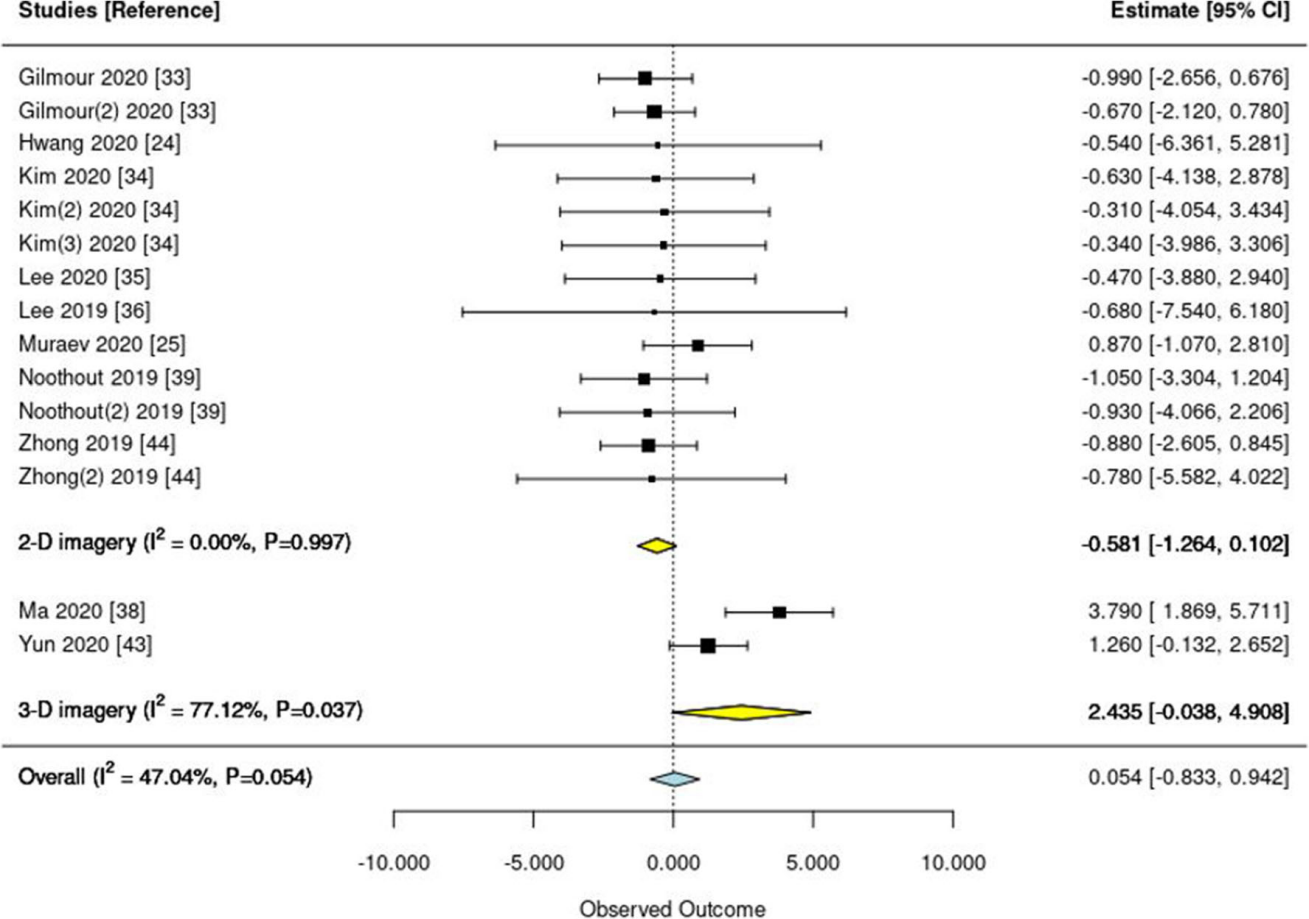
Forest plot of studies reporting the mean deviation from a 2-mm prediction error threshold. Squares indicate the mean deviation of each single study and lines the 95% confidence intervals (95% CI). Yellow and blue diamonds show the pooled subtotal (on 2-D and 3-D imagery) and overall estimates, respectively. I-square and the P value indicate heterogeneity. Studies are ordered according to year; if multiple test datasets were employed in the same study, the second or third is indicated accordingly (e.g., Noothout 2019 (2))

**Fig. 3:**
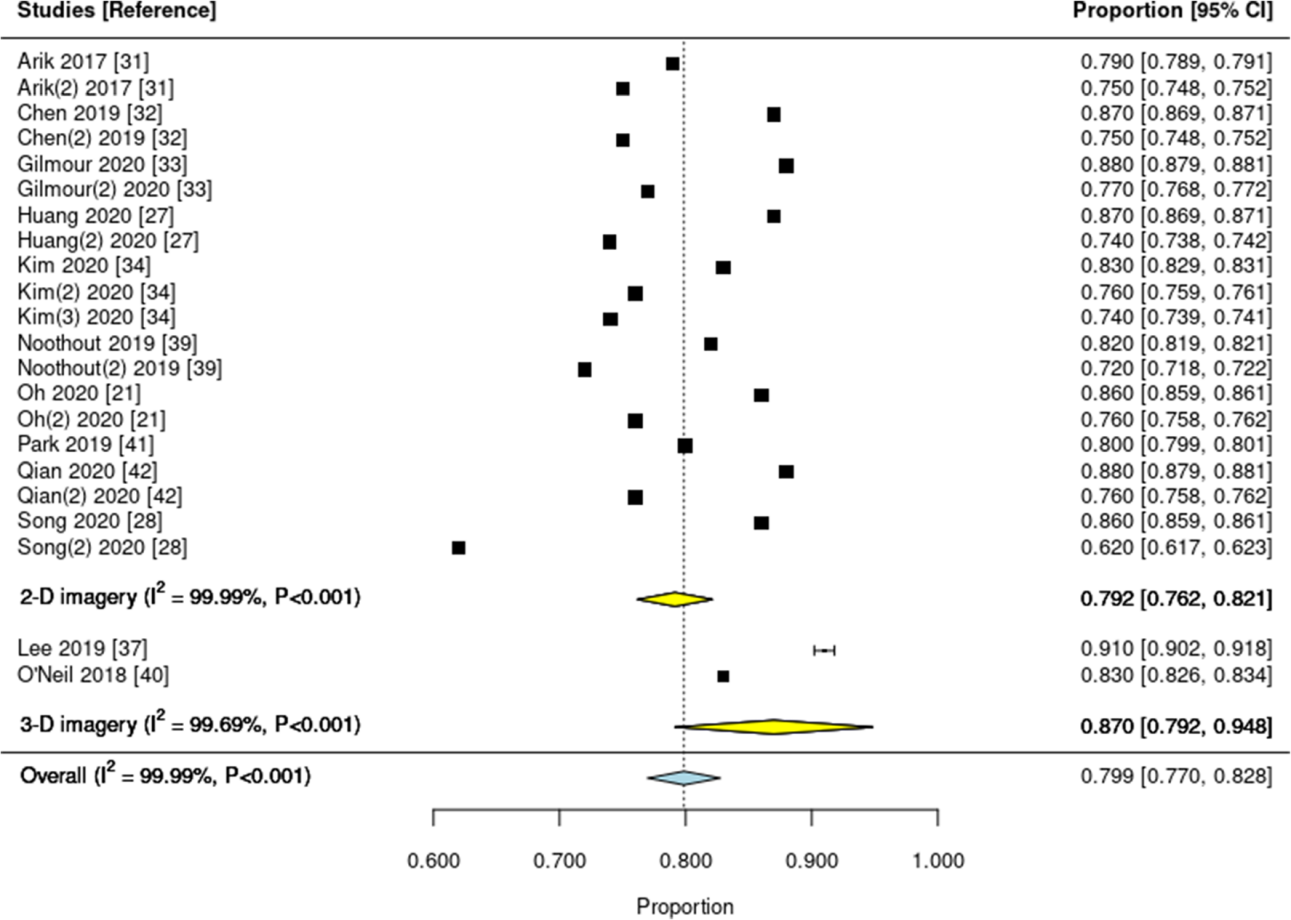
Forest plot of studies reporting the proportion of landmarks correctly predicted within a 2-mm prediction error threshold from the reference. Squares indicate the mean proportion found in each single study and lines the 95% confidence intervals (95% CI). Yellow and blue diamonds show the pooled subtotal (on 2-D and 3-D imagery) and overall estimates, respectively. I-square and the P value indicate heterogeneity. Studies are ordered according to year; if multiple test datasets were employed in the same study, the second or third is indicated accordingly (e.g., Arik 2017 (2))

Two meta-analyses were performed, one synthesizing the mean deviation from a 2-mm prediction error threshold (in mm) (Fig. 2) and one on the proportion of landmarks detected within this 2-mm threshold (Fig. 3). One study [33] reported the mean deviation, but not in mm but pixel. As this study would have introduced additional heterogeneity given the different outcome measure, it was not included in the first meta-analysis (on the mean deviation from the 2-mm threshold), but we could include it in the second meta-analysis (on the proportion of landmarks lying within the 2-mm threshold).

Regarding the mean deviation from a 2-mm prediction error, an overall number of 10 studies contributed 15 estimates (three studies had tested the DL model on 2 test datasets, one study on 3 test datasets). Of these, 8 studies (13 estimates) reported on 2-D radiographs, 2 on 3-D radiographs. Pooling revealed moderate heterogeneity (I^2^=47%; P=0.05), mainly due to differences between 2- and 3-D imagery. On 2-D imagery, predictions were largely below the 2-mm threshold (–0.581; 95 CI: –1264 to 0.102 mm), on 3-D imagery above the threshold (2.435; –0.038 to 4.908 mm). Overall, only 3 studies (1 on 2-D, 2 on 3-D images) had a mean deviation exceeding the 2-mm threshold (Fig. 2). The pooled deviation from the 2-mm error was 0.054 (–0.833 to 0.942). Meta-regression revealed no significant association between the mean deviation and the year of publication (P=0.494).

Regarding the proportion of landmarks detected with the 2-mm threshold, 12 studies with 22 estimates were included (20 estimates were for 2-D radiographs, 2 for 3-D radiographs); heterogeneity was high (I^2^=99%; P<0.001). The overall proportion was 0.799 (0.770 to 0.824); the proportion was lower for 2-D (0.792; 0.762 to 0.821) than 3-D (0.870; 0.792 to 0.948) imagery. Meta-regression on the year of publication did not reveal a significant association (P=0.916).

## Discussion

In contrast to most other dental radiographs, cephalograms are not only diagnosed qualitatively but also quantitatively through angular and linear measurements often in relation to reference planes (considered as stable structures) [40]. Most quantitative analyses are based on the identification of reference points which are either skeletal landmarks, e.g., the anterior nasal spine, virtual points such as the sella (middle of sella turcica), or constructed points like the gonion (crossing point between two lines) [8]. To yield meaningful results, the precise identification of the landmarks is crucial [41]. The present review evaluated studies using DL for landmark detection on 2-D or 3-D cephalometric imagery. Based on 19 included studies, we found DL accurate for this purpose; the majority of studies did not exceed a 2-mm prediction error threshold in mean, and the mean proportion of landmarks detected within this 2-mm threshold was 80%. The findings were largely consistent across studies; the most notable difference in accuracy was found between 2-D and 3-D images. However, direction of this difference was not consistent between our two outcomes—for the mean deviation from a 2-mm prediction error, 3-D images showed higher deviations, while for the proportion of predictions below this 2-mm threshold, 3-D images showed a higher proportion. Data on 3-D was generally sparse. The majority of studies showed high risk of bias and applicability concerns. This needs highlighting, as a number of software tools are by now already on the market—often with unclear scientific underpinning. The finding of high risk of bias and concerns in the current body of evidence is worrisome in this regard.

A number of our findings need to be discussed. First, the reported mean deviations were rather consistent across studies; the detected heterogeneity in our first meta-analysis was mainly due to differences between 2-D and 3-D imagery. The proportion of landmarks detected within the 2-mm threshold varied more markedly, with one study showing only 62% of landmarks achieving that threshold. When assessing which specific landmarks were prone to not being detected correctly, the porion, subspinale, gonion, articulare, and the anterior nasal spine were most often found to show larger deviations. However, these findings were not necessarily consistent across studies, and for these landmarks, DL did not necessarily perform worse than clinicians. Overall, and based on the two studies which evaluated human comparator groups against DL, we conclude that DL performs similar as regular clinicians [24] or even superior to inexperienced ones [30]. Generally, it is difficult to compare mean deviations between studies, as they largely depend on the test dataset: Even in the widely used and publicly available IEEE dataset, both the clinicians but also DL consistently performed worse on the test dataset 2 (containing 100 images) than dataset 1 (containing 150 images). Moreover, DL may not exceed experts’ accuracy but may obviously assist landmark detection for regular or experienced examiners. Training models on larger datasets may eventually help to even be as or more accurate than experts [42].

Second, data on 3-D imagery were sparse; only four studies employed DL for this purpose. A recent systematic review compared DL with knowledge-, atlas- and shallow-learning–based methods for 3-D landmark detection and concluded that DL was most accurate [13]. Given the paucity in data, however, it is difficult to strongly endorse DL for 3-D landmark detection at present. Generally, it should be considered that CBCT-based assessments will not be the rule for many orthodontic patients, but rather the exception, for example, when planning orthognatic surgery [9].

Third, we did not identify significant changes in accuracy in studies published in different years. One may expect more recent studies to show higher accuracies, as larger datasets, more powerful hardware, and more effective DL architectures might be available. Regarding the datasets, this was obviously not the case; the usage of the IEEE 2015 dataset was as common in 2020 as it was in 2017–2019. Moreover, it is likely that more powerful hard- or software can only be limitedly leveraged on datasets containing only a few hundred images like the IEEE 2015 one. Also, we did not identify a consistent evolution of the employed architectures and found only limited benefit of newer architectures (there was only one study on this issue, and this study found the accuracies of LeNet-5 and ResNet50 on the same 3-D test data to be similar) [23].

Fourth, the relevance of the test dataset (as discussed) was confirmed. The consistent difference in accuracy on the two test datasets of the IEEE 2015 challenge has been mentioned; an even more dramatic drop in accuracy was found when models were tested on a fully external dataset [36]. It is commendable that given the IEEE dataset composition, many studies had two test datasets. However, as all studies tested in this same dataset (and most also trained on this dataset), we likely have high comparability but limited generalizability. Future studies should aim to test DL models on broad data, demonstrating robustness and generalizability.

This review and the included studies have a number of limitations. First, we focused on DL for landmark detection; a comparison against other (semi-)automated landmarking methods has not been conducted. Second, we had to exclude a number of studies, e.g., those using DL for predicting skeletal anomalies (i.e., skipping landmark detection and analysis) or those which were unavailable in full text, likely losing some valuable data. Third, the included studies suffered from a range of risks of bias. Data selection yielded small and possibly nonrepresentative populations; the majority of studies employed the same dataset (meaning that all these studies can show their DL model to work on exactly this single test dataset, not on data from other populations). Regarding data representativeness, the overall evidence was highly limited; especially for 3-D imagery, scans in the test dataset usually stemmed from only few patients. The reference test (i.e., how the ground truth was established) was only sparsely described; in many studies, it was not exactly clear how the labels of one or more human annotator(s) eventually resulted in the training and test dataset. Some studies used only one expert as reference test, a decision which may be criticized given the wide variability in experts’ landmarking, as discussed. Any DL model trained on such dataset will be only as good as this single expert. Fourth, and as discussed, only few studies tested the developed DL models on truly independent datasets, e.g., from different centers, populations, or image generators, contributing to the limitations in generalizability. Fifth, it must be kept in mind that a location error of <2.0 mm may be acceptable for some, but not all landmarks: For example, the location error of the A and B point is usually large in the vertical and small in the horizontal plane, the latter being the important direction for determining the sagittal jaw relationship [8, 41]. In this direction and on these points, location errors of 1.9 mm would be considered inacceptable [8, 43] It should be also kept in mind that so-called stable reference structures are subject to variation, with dental experts remaining needed to critically assess AI findings [44] Last, the studies mainly employed accuracy estimates (this was partially the result of our inclusion criteria), while different outcome measures (deviations in mm, pixels, or proportions) were employed, which are not necessarily comparable. Further outcomes with relevance to clinicians, patients, or other stakeholders (like the impact of using a DL tool in clinical routine on diagnostic and treatment processes, their efficacy, safety, or efficiency) were not reported. Future studies should consider including a wider outcome set and aim to test DL applications comprehensively in other study designs and settings (e.g., observational studies in clinical care, randomized controlled trials). Also, it should be considered that the requirements toward AI-based cephalometric analyses may differ according to the resulting treatment decisions: Deviations acceptable when planning aligner treatments in Class I patients may be intolerable when planning surgical interventions, for instance.

## Conclusion

DL shows relatively high accuracy for detecting landmarks on cephalometric imagery. The majority of studies focused on 2-D imagery; data on 3-D imagery are sparse, but promising. There is heterogeneity in detection accuracy between landmarks, and it remains unclear if clinicians are similar, more or less accurate than DL for different landmarks. The overall evidence, while by large consistent, is of limited generalizability and robustness, and the true value of using DL in clinical care needs to be demonstrated.

## Supplementary Information

ESM 1 (DOCX 34 kb)
