# Supplementary material for: Deep learning for cephalometric landmark detection: systematic review and meta-analysis
Source: Clin Oral Investig. 2021 May 27;25(7):4299–309. doi: 10.1007/s00784-021-03990-w (PMC8310492; doi:10.1007/s00784-021-03990-w)
Supplement: Supplementary file 1 — (DOCX 34 kb) [file 784_2021_3990_MOESM1_ESM.docx]

**Appendix**

Table S1: Excluded studies

| **Study** | **Excluded** |
| --- | --- |
| [1] | Testing software, unclear how trained |
| [2] | No deep learning |
| [3] | No landmark detection |
| [4] | No deep learning |
| [5] | No deep learning |
| [6] | Results not synthesizable |
| [7] | No landmark detection |
| [8] | No deep learning |
| [9] | No deep learning |
| [10] | No landmark detection |
| [11] | Unavailable |
| [12] | Unavailable |
| [13] | No deep learning |
| [14] | Unavailable |
| [15] | Unavailable |
| [16, 17] | Results not synthesizable |
| [18] | No deep learning |
| [19] | No deep learning |
| [20] | Unavailable |
| [21] | Results not synthesizable |

**References for the appendix**

[1] P. Meriç, J. Naoumova, Web-based Fully Automated Cephalometric Analysis: Comparisons between App-aided, Computerized, and Manual Tracings, Turk J Orthod 33(3) (2020) 142-149.

[2] A. Gupta, O.P. Kharbanda, V. Sardana, R. Balachandran, H.K. Sardana, A knowledge-based algorithm for automatic detection of cephalometric landmarks on CBCT images, Int J Comput Assist Radiol Surg 10(11) (2015) 1737-52.

[3] I. Kim, D. Misra, L. Rodriguez, M. Gill, D.K. Liberton, K. Almpani, J.S. Lee, S. Antani, Malocclusion Classification on 3D Cone-Beam CT Craniofacial Images Using Multi-Channel Deep Learning Models(), Annu Int Conf IEEE Eng Med Biol Soc 2020 (2020) 1294-1298.

[4] Y. Jiang, G. Song, X. Yu, Y. Dou, Q. Li, S. Liu, B. Han, T. Xu, The application and accuracy of feature matching on automated cephalometric superimposition, BMC medical imaging 20(1) (2020) 31.

[5] J. Montúfar, M. Romero, R.J. Scougall-Vilchis, Automatic 3-dimensional cephalometric landmarking based on active shape models in related projections, American journal of orthodontics and dentofacial orthopedics : official publication of the American Association of Orthodontists, its constituent societies, and the American Board of Orthodontics 153(3) (2018) 449-458.

[6] H.S. Yun, T.J. Jang, S.M. Lee, S.H. Lee, J.K. Seo, Learning-based local-to-global landmark annotation for automatic 3D cephalometry, Physics in medicine and biology 65(8) (2020) 085018.

[7] H.J. Yu, S.R. Cho, M.J. Kim, W.H. Kim, J.W. Kim, J. Choi, Automated Skeletal Classification with Lateral Cephalometry Based on Artificial Intelligence, Journal of dental research 99(3) (2020) 249-256.

[8] C.W. Wang, C.T. Huang, M.C. Hsieh, C.H. Li, S.W. Chang, W.C. Li, R. Vandaele, R. Marée, S. Jodogne, P. Geurts, C. Chen, G. Zheng, C. Chu, H. Mirzaalian, G. Hamarneh, T. Vrtovec, B. Ibragimov, Evaluation and Comparison of Anatomical Landmark Detection Methods for Cephalometric X-Ray Images: A Grand Challenge, IEEE Trans Med Imaging 34(9) (2015) 1890-900.

[9] C. Lindner, C.-W. Wang, C.-T. Huang, C.-H. Li, S.-W. Chang, T.F. Cootes, Fully Automatic System for Accurate Localisation and Analysis of Cephalometric Landmarks in Lateral Cephalograms, Scientific reports 6(1) (2016) 33581.

[10] J. Zhang, M. Liu, D. Shen, Detecting Anatomical Landmarks From Limited Medical Imaging Data Using Two-Stage Task-Oriented Deep Neural Networks, IEEE Trans Image Process 26(10) (2017) 4753-4764.

[11] M. Ed-Dhahraouy, H. Riri, M. Ezzahmouly, F. Bourzgui, A. El Moutaoukkil, A new methodology for automatic detection of reference points in 3D cephalometry: A pilot study, International orthodontics 16(2) (2018) 328-337.

[12] S. Nishimoto, Y. Sotsuka, K. Kawai, H. Ishise, M. Kakibuchi, Personal Computer-Based Cephalometric Landmark Detection With Deep Learning, Using Cephalograms on the Internet, The Journal of craniofacial surgery 30(1) (2019) 91-95.

[13] B.C. Neelapu, O.P. Kharbanda, V. Sardana, A. Gupta, S. Vasamsetti, R. Balachandran, H.K. Sardana, Automatic localization of three-dimensional cephalometric landmarks on CBCT images by extracting symmetry features of the skull, Dento maxillo facial radiology 47(2) (2018) 20170054-20170054.

[14] M. Zeng, Z. Yan, S. Liu, Y. Zhou, L. Qiu, Cascaded convolutional networks for automatic cephalometric landmark detection, Med Image Anal 68 (2020) 101904.

[15] E.N.D. Goutham, S. Vasamsetti, P.V.V. Kishore, H.K. Sardana, AUTOMATIC LOCALIZATION OF LANDMARKS IN CEPHALOMETRIC IMAGES Via MODIFIED U-Net, 2019 10th International Conference on Computing, Communication and Networking Technologies (ICCCNT), 2019, pp. 1-6.

[16] N. Torosdagli, M. McIntosh, D. Liberton, P. Verma, M. Sincan, W. Han, J. Lee, U. Bagci, Relational Reasoning Network (RRN) for Anatomical Landmarking, arXiv:1904.04354v1 (2019).

[17] N. Torosdagli, D.K. Liberton, P. Verma, M. Sincan, J.S. Lee, U. Bagci, Deep Geodesic Learning for Segmentation and Anatomical Landmarking, IEEE Transactions on Medical Imaging (2018) 1-14.

[18] A. Gupta, O.P. Kharbanda, V. Sardana, R. Balachandran, H.K. Sardana, Accuracy of 3D cephalometric measurements based on an automatic knowledge-based landmark detection algorithm, International Journal of Computer Assisted Radiology and Surgery 11(7) (2016) 1297-1309.

[19] M. Codari, M. Caffini, G.M. Tartaglia, C. Sforza, G. Baselli, Computer-aided cephalometric landmark annotation for CBCT data, Int J Comput Assist Radiol Surg 12(1) (2017) 113-121.

[20] J. Qian, M. Cheng, Y. Tao, J. Lin, H. Lin, CephaNet: An Improved Faster R-CNN for Cephalometric Landmark Detection, 2019 IEEE 16th International Symposium on Biomedical Imaging (ISBI 2019), 2019, pp. 868-871.

[21] F. Kunz, A. Stellzig-Eisenhauer, F. Zeman, J. Boldt, Artificial intelligence in orthodontics : Evaluation of a fully automated cephalometric analysis using a customized convolutional neural network, Journal of orofacial orthopedics = Fortschritte der Kieferorthopadie : Organ/official journal Deutsche Gesellschaft fur Kieferorthopadie 81(1) (2020) 52-68.
